# Assessing predictive performance of supervised machine learning algorithms for a diamond pricing model

**DOI:** 10.1038/s41598-023-44326-w

**Published:** 2023-10-12

**Authors:** Samuel Njoroge Kigo, Evans Otieno Omondi, Bernard Oguna Omolo

**Affiliations:** 1 Institute of Mathematical Sciences, Strathmore University, P.O Box 59857-00200 Nairobi, Kenya; 2 African Population and Health Research Center, P.O Box 10787-00100 APHRC Campus, Kitisuru, Nairobi, Kenya; 3 Division of Mathematics and Computer Science, University of South Carolina–Upstate, Hodge Center 223 800 University Way, Spartanburg, SC 29303 USA; 4 Faculty of Health Sciences, School of Public Health, University of the Witwatersrand, Johannesburg, South Africa

**Keywords:** Climate sciences, Mathematics and computing

## Abstract

This study conducted a comprehensive analysis of multiple supervised machine learning models, regressors and classifiers, to accurately predict diamond prices. Diamond pricing is a complex task due to the non-linear relationships between key features such as carat, cut, clarity, table, and depth. The analysis aimed to develop an accurate predictive model by utilizing both regression and classification approaches. To preprocess the data, the study employed various techniques. The work addressed outliers, standardized the predictors, performed median imputation of missing values, and resolved multicollinearity issues. Equal-width binning on the cut variable was performed to handle class imbalance. Correlation-based feature selection was utilized to eliminate highly correlated variables, ensuring that only relevant features were included in the models. Outliers were handled using the inter-quartile range method, and numerical features were normalized through standardization. Missing values in numerical features were imputed using the median, preserving the integrity of the dataset. Among the models evaluated, the RF regressor exhibited exceptional performance. It achieved the lowest root mean squared error (RMSE) of 523.50, indicating superior accuracy compared to the other models. The RF regressor also obtained a high R-squared ($\text {R}^2$) score of 0.985, suggesting it explained a significant portion of the variance in diamond prices. Furthermore, the area under the curve with RF classifier for the test set was 1.00 $\, (100\%)$, indicating perfect classification performance. These results solidify the RF’s position as the best-performing model in terms of accuracy and predictive power, both in regression and classification. The MLP regressor showed promising results with an RMSE of 563.74 and an $\text {R}^2$ score of 0.980, demonstrating its ability to capture the complex relationships in the data. Although it achieved slightly higher errors than the RF regressor, further analysis is needed to determine its suitability and potential advantages compared to the RF regressor. The XGBoost Regressor achieved an RMSE of 612.88 and an $\text {R}^2$ score of 0.972, indicating its effectiveness in predicting diamond prices but with slightly higher errors compared to the RF regressor. The Boosted Decision Tree Regressor had an RMSE of 711.31 and an $\text {R}^2$ score of 0.968, demonstrating its ability to capture some of the underlying patterns but with higher errors than the RF and XGBoost models. In contrast, the KNN regressor yielded a higher RMSE of 1346.65 and a lower $\text {R}^2$ score of 0.887, indicating its inferior performance in accurately predicting diamond prices compared to the other models. Similarly, the Linear Regression model performed similarly to the KNN regressor, with an RMSE of 1395.41 and an $\text {R}^2$ score of 0.876. The Support Vector Regression model showed the highest RMSE of 3044.49 and the lowest $\text {R}^2$ score of 0.421, indicating its limited effectiveness in capturing the complex relationships in the data. Overall, the study demonstrates that the RF outperforms the other models in terms of accuracy and predictive power, as evidenced by its lowest RMSE, highest $\text {R}^2$ score, and perfect classification performance. This highlights its suitability for accurately predicting diamond prices. The study not only provides an effective tool for the diamond industry but also emphasizes the importance of considering both regression and classification approaches in developing accurate predictive models. The findings contribute valuable insights for pricing strategies, market trends, and decision-making processes in the diamond industry and related fields.

## Introduction

Diamond, renowned as the planet’s hardest substance, has gained enduring recognition for its exquisite beauty as a precious gemstone. As of 2019, cumulative diamond extraction worldwide was estimated at 142 million carats. Major diamond-producing countries, including Australia, Canada, the Democratic Republic of Congo, Botswana, South Africa, and Russia, have played pivotal roles in meeting global demand. Notably, the world’s diamond reserves are estimated to hold approximately 1.2 billion carats, with Russia boasting the largest share valued at around 650 million carats. The abundance of these reserves underscores Russia’s influential position in the diamond market^1^. In 2020, global diamond jewelry sales were 68 billion (in US dollars) according to^2^, with the United States accounting for 35 billion (in US dollars) of that amount^3^. In 2019, the United States had the biggest demand for polished diamonds, totaling 12.8 billion (in US dollars)^4^. The United States, China, India, Japan, and the Persian Gulf region are the top five markets for diamonds, according to^5^.

Diamonds are a very unique consumer product. It is the hardest mineral i.e. 58 times harder than any other mineral^6^, thus used in various machines and other types of equipment for cutting and slicing. Diamonds demand, on the other hand, is not directly tied to such inherent features, but rather to their perceived value as rare and expensive items^5^. It is one of the gemstones on which more money is spent than any other combined gemstone. The diamond gains popularity because it has an optical property^6^. Other factors include its durability, custom, fashion, and aggressive marketing by diamond producers^7^. Due to its non-linearity and fluctuating time series behavior, forecasting the prices of diamond is a difficult task^5^. Banks prefer to invest in precious metals such as gold and diamond owing to their their unique features and great market demand. As a client, you’re constantly unsure when it’s the best moment to invest in, buy, or sell valuable items like gold and diamonds^8^. When it comes to generating the greatest profit out of an investment and the least expense out of a purchase made for the above-mentioned products, pricing is extremely important to buyers and investors.

The 4Cs—*Cut*, *Carat*, *Color*, and *Clarity*—were introduced by the Gemological Institute of America (GIA) in the 1950s and are the most well-known attributes of diamonds. The 4Cs describe each diamond’s distinct characteristics and have a significant impact on diamond prices. Three of the four Cs have a lengthy history: *carat weight*, *color*, and *clarity* were all utilized in the original diamond grading system over 2,000 years ago in India^5^. The dimensions of a diamond’s cut determine how efficiently it reflects light. On a scale of fair to ideal, the cut perfection is classified. One of the main components of the *cut* variable is the degree of perfection achieved by the cutting and polishing process. This is a complicated variable that includes, among other things, the stone’s symmetry and adherence to local market-specific standards regarding the stone’s proportions and the presence or absence of specific features such as an ID number engraved in the stone’s girdle, the girdle’s faceting, and so on^9^.

The cut of a diamond also has three other characteristics: brilliance, or the amount of light reflected from it; fire, or the dispersion of light into the colors of the spectrum; and scintillation, or the flashes of glitter that occur when a diamond is moved around^5^. According to the International Gem Society, out of 4Cs, the *cut* is the most important attribute of a diamond^10^. Chu^11^ asserts that optimal cut should neither be too deep nor too shallow for it will impede the trajectory of light and thereby the brilliance or “fire” of a diamond stone. Blue Nile, one of the largest online diamond retailers, asserts that *cut* has the biggest effect on the sparkle, and even with perfect *color* and *clarity*, a poorly cut diamond will look dull^12^. In addition to 4Cs, there are many other attributes of diamonds such as *length*, *width*, *height*, *table*, etc. To better understand how such complex features influence diamond prices, the study proposes application of supervised machine learning algorithms (SMLAs). SMLAs afford the advantage of capturing non-linearity relationships in a given dataset.

The diamond trade industry presents buyers and investors with numerous challenges in accurately estimating the price of diamond stones. The complexity arises from the non-linear nature of factors that influence diamond prices, including carat, cut, clarity, table, and depth. Unlike other commodities, diamonds could not be valued solely based on weight or purity. Instead, their worth is heavily influenced by these intricate factors, making the pricing process much more challenging. Moreover, the unique characteristics of diamond stones, such as their shapes, sizes, and clarity, further complicate the task of estimating their value. Traditionally, the diamond pricing process has relied on expert knowledge and subjective assessments, which could lead to inconsistencies and disparities in pricing. *Rapaport list*, price list that quote Rapaport’s opinion of high cash asking prices generally accepted as high enough to serve as the initial starting point for negotiations, has been used in classical diamond evaluation. The Rapaport list prices are almost always higher than actual dealer transaction prices, which tend to trade at discounts to the list. Final transaction prices are the result of negotiations between buyer and seller thus being difficult to predict based only on the Rapaport price list^9^.

Various models and methods have been developed to address these challenges. For instance, the 4Cs (carat, cut, clarity, and color) are widely used as a framework for evaluating diamonds, with each factor assigned a specific weight in the pricing equation. However, these models often fail to capture the intricate relationships between these factors and the final diamond price, resulting in limitations and inaccuracies. All these factors combined make it challenging for buyers and investors to predict the price of diamond stones with precision, which ultimately affects their ability to make informed investment decisions in the diamond trade industry^13^. To overcome these limitations and enhance pricing accuracy, the application of supervised machine learning algorithms has gained attention in the diamond trade industry. Machine learning algorithms have the potential to capture complex patterns and relationships within large datasets, enabling the development of robust diamond pricing models. By training on historical data that includes various diamond characteristics and their corresponding prices, these algorithms can learn to make accurate predictions for new diamond stones.

This study aims to assess the predictive performance of eight supervised machine learning algorithms, including Multiple Linear Regression, Linear Discriminant Analysis, eXtreme Gradient Boosting, Random Forest, k-Nearest Neighbors, Support Vector Machines, Boosted Regression and Classification Trees, and Multi-Layer Perceptron for accurate diamond price estimation. These algorithms were selected to represent diverse approaches and techniques, considering their popularity, versatility, and documented success in various domains. The selection was informed by a review of relevant literature on diamond pricing and machine learning applications, aiming to evaluate their predictive performance specifically for a diamond pricing model. However, other algorithms may have been considered but were not included in this study. Future research could explore the inclusion of additional algorithms to further enhance insights into diamond price estimation. The findings of this study would contribute to the development of more reliable and objective pricing methods in the diamond trade industry. The implications of this research are significant for industry practitioners and stakeholders. A more accurate diamond pricing model could benefit buyers and investors by providing them with objective and transparent pricing information. It could also assist in mitigating the disparities and inconsistencies that currently exist in the industry. Furthermore, the adoption of machine learning algorithms in diamond pricing could streamline the pricing process, saving time and resources for industry professionals.

Contemporary statistical analysis is characterized with the evolution of machine and deep learning algorithms. Ahmed et al.^14^ and Kampichler et al.^15^ observe that these algorithms have been empirically proven to be serious contenders to classical statistical models in dealing with high dimensional data that are often non-linear and do not meet the assumptions of conventional statistical procedures. This aspect thus affirms the decision to employ them in this work for diamond price prediction and classification since they offer promising opportunities to enhance pricing accuracy. The aforementioned assertions are based on various predictive performance metrics including Precision, Accuracy, Kappa, Recall, F-Measure, ROC, RMSE, MAE, R-squared and computational aspects such as speed and run time, among others. The knowledge of best performing model(s) is imperative in refocusing the modeler’s time, effort and other resources to only potential candidate models in machine learning and pattern recognition.

In classical statistics, modeling complex non-linear relationships was the biggest drawback until 1980’s where advancement in computing technology permitted non-linear modeling^16^. The explosion of ‘Big Data’ has seen the release of over $90\%$ of the current world data in just about the past four years^17^. There exists a pressing need by businesses, governments and researchers to draw meaningful insights out of these overwhelming amounts of data in making smarter decisions. Businesses are actually beginning to view data as a cocktail of fresh opportunities or as crude oil that needs to be refined using cutting-edge knowledge and skills, i.e., by gaining insight into the engineering process that underlies data and finding the hidden patterns that will guide valuable iterations of investment decisions. It is clear that the next battleground for business rivalry and other commercial underpinnings is data.

The new epoch of Big Data is redefining statistical learning applications on supervised and unsupervised modeling and prediction. Osisanwo et al.^18^ postulates that this tendency could be traced back to the advancement of Smart and Nano technologies, which has sparked interest in uncovering hidden patterns in data, both structured and unstructured, to derive value. Further, increase in the freely available and user-friendly statistical softwares such as *R* and *Python* has provided an upthrust to machine learning innovations in modeling. This research explores the use of supervised machine learning algorithms to investigate the relationship between diamond physical qualities and diamond prices in order to establish the extent to which the latter are determined by the former.

Diamonds are one of the most valuable and expensive gems in the world, and their price is determined by various factors such as carat weight, color, clarity, and cut. The cut itself contains three key aspects: brilliance, dispersion, and scintillation, all of which attract the attention of the diamond market’s major players. Traditional methods of diamond price prediction, as earlier discussed, rely on expert assessment, which could be subjective and time-consuming. As such, there is a need for more accurate and efficient methods for diamond price prediction. Supervised machine learning algorithms have been increasingly used in predicting diamond prices due to their ability to learn from past data and make accurate predictions. However, the predictive performance of these algorithms varies depending on the algorithm and the dataset used.

Previous research on diamond price prediction models in machine learning has explored various algorithms and approaches, considering factors such as diamond size, physical attributes, and pricing determinants. However, these studies have some limitations, including overlooking the importance of classification, inadequate model comparisons, and the omission of relevant evaluation metrics. In contrast, the this study addresses these gaps by conducting a comprehensive analysis of multiple supervised machine learning models, encompassing both regressors and classifiers, to accurately predict diamond prices. The study preprocesses the data using various techniques to handle outliers, standardize predictors, impute missing values, and resolve multicollinearity. It employs regression and classification approaches and evaluates the models based on evaluation metrics such as RMSE, R-squared, and AUC. The results highlight the Random Forest (RF) regressor as the best-performing model, demonstrating superior accuracy, predictive power, and perfect classification performance. The study also explores other models such as MLP, XGBoost, Boosted Decision Tree, KNN, and Linear Regression, providing insights into their performance. By showcasing the RF’s effectiveness and emphasizing the importance of considering both regression and classification, this study contributes valuable insights for the diamond industry and decision-making processes in related fields.

Therefore, the aim of this research is to select the most accurate supervised machine/deep learning algorithm for diamond price prediction, specifically focusing on both classification and regression approaches. Accurate forecasting of diamond prices is crucial for the diamond industry, as it allows stakeholders to make informed decisions, manage risks, and optimize profits. As such, the development of improved forecasting methods using machine and deep learning techniques represents an important area of research for the industry. In this study, we will employ machine learning algorithms and ensemble methods, including boosting and bootstrapped aggregation, to predict diamond prices. Boosting has been shown to ameliorate classifier performance^19^. However, despite their strong empirical performance, most model comparison studies have not applied ensemble approaches^20^.

Both classification and regression-based models will be used to predict diamond prices. For the classification-based model, we will perform equal-width binning on the cut variable since the range of the cut values is not evenly distributed. In this technique, each attribute’s range is divided into a fixed number of equal magnitude bins and values are converted into bin numbers to improve interpretability^21^. Equal-width binning ensures that each bin has the same range of values, which helps to maintain the balance and equal representation of the data in each bin^22^. This is particularly important when building predictive models, as it helps to prevent the model from being biased towards certain segments of the data.

In the context of predicting diamond prices, the cut variable has five levels: Fair, Good, Very Good, Premium, and Ideal. To facilitate the machine learning model’s ability to accurately predict diamond prices, we will perform binning on the cut variable. Binning is a commonly used technique to transform continuous variables into discrete ones, and in this case, we will group diamonds into categories based on their cut. This technique helps to minimize the impact of outliers and extreme values^23^, which may skew the results of statistical analyses.

By grouping diamonds into categories based on their cut, the authors could focus on identifying patterns and relationships within each category, enabling the machine learning model to more accurately predict diamond prices. The resulting categories will serve as inputs to the machine learning model, and by grouping similar diamonds together, we could facilitate the model’s ability to identify patterns in the data and generate more precise predictions.

The study will evaluate the performance of our regression-based models using metrics such as RMSE and R-squared, while for the classification-based models, we will use accuracy, precision, recall, ROC and F-measure. Through this study, we aim to provide insights into the potential of supervised machine learning for diamond price prediction and develop more accurate and efficient models.

This paper makes the following significant contributions to the field of diamond pricing:

1. The study presents an accurate predictive model for diamond prices by employing a comprehensive analysis of multiple supervised machine learning models. By utilizing both regression and classification approaches, the researchers develop a robust predictive model that considers the non-linear relationships between diamond features such as carat, cut, clarity, table, and depth. The use of Random Forest as the most optimal algorithm for both regression and classification tasks demonstrates its effectiveness as a predictive tool for diamond pricing.
2. The study provides valuable insights into pricing strategies and market trends in the diamond industry. Through exploratory data analysis and the analysis of various variables, including categorical variables such as color, cut, and clarity, the researchers identify patterns in the data that enable accurate predictions of diamond prices based on specific cut categories. This information can inform pricing strategies and help businesses adapt to market trends, ultimately enhancing their competitiveness in the industry.
3. The publication highlights the importance of considering both regression and classification approaches when developing predictive models for diamond pricing. By utilizing regression analysis, the researchers capture the continuous relationship between diamond prices and various features, while the classification approach, particularly for the cut variable, enables the prediction of mean prices for each cut category. This comprehensive approach provides a deeper understanding of the factors influencing diamond prices and offers a more accurate prediction of prices based on different characteristics, contributing to more informed decision-making processes in the diamond industry.

This paper is structured as follows. In Sect. "Related literature", we review the related literature about diamond price prediction, Sect. "Methods and materials" gives detailed information and description of research methodology that was applied. The focus of Sect. "Results" is data analysis and interpretation of results. It entails exploratory analysis, descriptive statistics and correlation analysis to estimate the relationship between variables. The study findings are interpreted and discussed in Sect. "Discussion" in relation to the research problem being investigated, and the conclusion is presented in Sect. "Conclusion".

## Related literature

Alsuraihi et al.^13^ aimed to develop an algorithm for accurate diamond price estimation, considering the diverse range of diamond sizes and other important factors. Various machine learning methods, including Linear Regression, Random Forest Regression, Polynomial Regression, Gradient Descent, and Neural Network, were employed to predict diamond prices. After training and analyzing multiple models, it was found that Random Forest Regression performed the best, with MAE and RMSE values of 112.93 and 241.97, respectively. However, the study failed to consider the significant class imbalance present in the dataset, rendering Random Forest inappropriate for such cases. Additionally, the study neglected the important factor of diamond classification, particularly the impact of the diamond cut on pricing. Augmenting regression results from Random Forest Classification could have enhanced the study’s findings.

Mamonov and Triantoro^5^ investigated the relationship between diamond physical features and pricing in the context of e-commerce, aiming to identify the influence of physical attributes on diamond prices. The primary determinants of diamond prices were found to be weight, color, and clarity. Decision Forest, Boosted Decision Tree, and Artificial Neural Network were employed as prediction data mining approaches, considering the continuous interval target variable with a ratio scale of diamond prices. Decision Forest yielded the lowest MAE of 5.8% when analyzing the complete dataset. When focusing on diamonds with a carat range of 0.2–2.5, ANN achieved an MAE of 8.2%, outperforming other techniques. However, the study overlooked the inclusion of other promising prediction data mining approaches, such as XGBoost, which have demonstrated good outcomes in model comparison studies. Evaluation criteria like $\text {R}^2$ and RMSE were not utilized. Furthermore, the study failed to consider the importance of diamond cut in the analysis, which significantly affects market value.

Pandey et al.^8^ addressed the challenge of forecasting future values of precious metals like gold and diamonds using ensemble approaches in combination with feature selection techniques. To mitigate over-fitting and under-fitting issues, a hybrid model combining Random Forest and Principal Component Analysis (PCA) was proposed. The study demonstrated that Random Forest outperformed Linear Regression with a mean accuracy of 0.9730 versus 0.8695. Additionally, Random Forest Regression with Chi-Square feature selection, using the five best features, achieved the highest accuracy of 0.9754 compared to 0.8663 for Linear Regression. However, the study did not provide a logical performance comparison with other high-performing machine learning models like MLP and XGBoost, which could address over-fitting and identify relevant features using variable importance. Moreover, the subjective implementation of PCA stifled statistical truth and independence, while essential evaluation metrics like $\text {R}^2$ and RMSE were not employed to validate the results.

Sharma et al.^7^ aimed to present supervised machine learning algorithms for predicting diamond prices. The study compared eight alternative supervised models, including Linear Regression, Lasso Regression, Ridge Regression, Decision Tree, Random Forest, ElasticNet, AdaBoost Regressor, and Gradient-Boosting Regressor, to identify the best-performing model. Random Forest exhibited superior performance according to the research, achieving an $\text {R}^2$ score of 0.9793 when the dataset was split into 80% for training and 20% for testing. However, the study overlooked the comparison of Random Forest with other novel machine learning algorithms like XGBoost or deep learning algorithms such as MLP. The importance of classification in pricing, particularly considering the significance of the diamond cut, was dismissed. Additionally, the study lacked the assessment of multiple regression metrics, such as MAE and RMSE, to provide a comprehensive evaluation of the results.

Mihir et al.^6^ addressed the challenge of predicting diamond prices by training machine learning models using various attributes. Algorithms such as Linear Regression, Support Vector Regression, Decision Trees, Random Forest Regression, kNeighbors Regression, CatBoost Regression, Huber Regression, Extra Tree Regression, Passive Aggressive Regression, Bayesian Regression, and XGBoost Regression were utilized. CatBoost Regression emerged as the most suitable algorithm for diamond price prediction, achieving the highest $\text {R}^2$ score of 0.9872 with comparatively lower RMSE and MAE values. However, the study acknowledged the need to incorporate additional factors such as shape, table value, polish, and symmetry to enhance the accuracy of predictions.

Chu^11^ aimed to build a pricing model for diamond stones, considering different degrees of clarity, color, and carat weight. The study employed Multiple Linear Regression (MLR) to predict diamond prices, considering carat, color, clarity, and certification factors. The resulting model achieved an $\text {R}^2$ value of 0.972. However, the study overlooked the non-linear relationship between caratage and price, which could have been addressed by employing machine learning models instead of MLR. Additionally, the analysis did not include other significant variables that could impact diamond pricing.

Scott and Yelowitz^24^ examined diamond prices in the context of commodities consumed for social status and intrinsic value. The study collected data from online diamond sellers and empirically investigated factors affecting diamond prices. Carat weight, color, cut, and clarity were considered in determining the logarithm of price. The study reported adjusted $\text {R}^2$ values of 0.889, 0.898, and 0.937 for Blue Nile, Union Diamond, and Amazon listed diamonds, respectively. However, considering the non-linear nature of the relationship between diamond attributes and price, the study should have explored the application of machine learning algorithms. Moreover, the absence of error measurements such as RMSE limited the validation of the findings

## Methods and materials

### Data type and source

**Figure 1:**
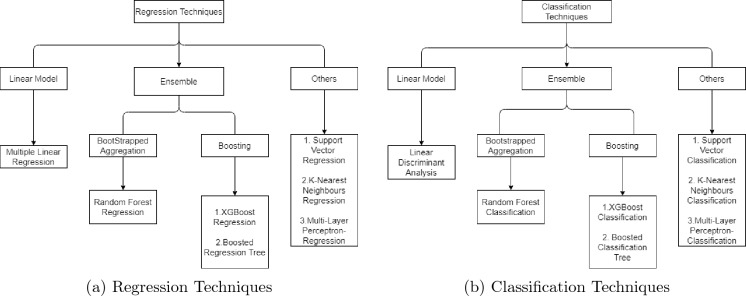
Description of the classifications of the SML models.

The diamond dataset used in this study was sourced from Kaggle, a well-known platform for data science enthusiasts. The dataset contains information on approximately 53,000 diamonds sold by a US-based retailer between 2008 and 2018. The data was collected by the retailer and includes attributes such as carat weight, cut, color, clarity, and price^25^. It is important to note that while the dataset provides valuable insights into the diamond industry, it is limited to diamonds sold by a single retailer in the United States. Therefore, caution must be exercised when generalizing the findings of this study to other regions and retailers. The data was divided into three sets in the following way: 56% for training, 24% for validation, and 20% for testing. This partitioning is a widely used and recommended practice in machine learning modeling. By doing this, we ensure that the model is trained on a substantial amount of data (the training set), while still having enough data to assess the model’s performance (the validation set) and evaluate its ability to generalize to new data (the test set). All of the models under consideration were subjected to a k-fold cross validation, with k set to 10. The study proposes SML models as outlined in Fig. 1. Figures 1a,b show regression and classification techniques, respectively.

### Selected algorithms’ summary

*Linear Discriminant Analysis (LDA)*

- *Advantages* Can handle multiclass classification problems, assumes multivariate normal distributions which may be appropriate for certain cases, provides dimensionality reduction by projecting data onto a lower-dimensional space.
- *Disadvantages* Assumes equal covariance matrices for different cut classes, may not perform well if the underlying assumptions are violated.

*eXtreme Gradient Boosting (XGBoost)*

- *Advantages* Handles complex relationships and interactions between predictors and diamond price, performs well on large datasets, provides feature importance ranking, handles missing data.
- *Disadvantages* Requires careful parameter tuning, may be computationally expensive and time-consuming.

*Random Forest (RF)*

- *Advantages* Reduces overfitting and captures variable interactions, handles high-dimensional data, provides feature importance ranking, robust to outliers and missing data.
- *Disadvantages* Less interpretable than individual decision trees, may be sensitive to noisy data, can be computationally expensive for large datasets.

*k-Nearest Neighbors (k-NN)*

- *Advantages* Simple and intuitive, can handle nonlinear relationships, no assumptions about the data distribution, can be effective with small to medium-sized datasets.
- *Disadvantages* Sensitive to the choice of *k*, computationally expensive for large datasets, requires proper scaling and handling of missing data.

*Support Vector Machines (SVM)*

- *Advantages* Effective in high-dimensional spaces, can handle nonlinear relationships through kernel functions, works well with small to medium-sized datasets, robust to outliers.
- *Disadvantages* Limited scalability for large datasets, sensitive to the choice of kernel and hyperparameters, binary classification inherently, needs to be extended for multiclass problems.

*Boosted Regression and Classification Trees (BCRT)*

- *Advantages* Handles nonlinear relationships, can capture complex interactions and non-additive effects, less prone to overfitting than individual decision trees, provides feature importance ranking.
- *Disadvantages* Requires careful parameter tuning, computationally expensive, may overfit if the number of trees is too high.

*Multi-Layer Perceptron (MLP)*

- *Advantages* Can learn complex relationships between predictors and diamond price, handles nonlinear patterns, can work with both numerical and categorical features, powerful representation learning.
- *Disadvantages* Requires careful tuning of architecture and hyperparameters, prone to overfitting if not regularized properly, computationally expensive for large datasets, lacks interpretability.

### Multiple linear regression (MLR)

We consider this model when the study variable involves more than one predictor variables. Here, the relationship is important in that it allows the mean function *E*(*y*) to depend on more than one predictor variables and to assume shapes other than straight line^26^. Given the model as:

$$\begin{aligned} y = & \,\beta _{0} + \beta _{1} X_{1} + \beta _{2} X_{2} + \ldots + \beta _{k} X_{k} + \varepsilon {\mkern 1mu} , \\ {\text{price}} = \, & \beta _{0} + \beta _{1} {\text{carat}} + \beta _{2} {\text{cut}} + \beta _{3} {\text{color}} + \beta _{4} {\text{clarity}} + \beta _{5} {\text{total depth}} + \beta _{6} {\text{table}} + \beta _{7} {\text{length}} \\ & + \beta _{8} {\text{width}} + \beta _{9} {\text{depth}} + \varepsilon {\mkern 1mu} , \\ \end{aligned}$$

now given that the $n-tuples$ of observations follow the same model, below is satisfied:

1 $$\begin{aligned} \left. \begin{array}{ccc} \text {price}_1&{}=&{}\beta _0+\beta _1\text {carat}_{1}+\beta _2\text {cut}_{1}+\dots +\beta _9\text {depth}_{1}+\varepsilon _1,\\ \text {price}_2&{}=&{}\beta _0+\beta _1\text {carat}_{2}+\beta _2\text {cut}_{2}+\dots +\beta _9\text {depth}_{2}+\varepsilon _2,\\ \vdots \\ \text {price}_{n}&{}=&{}\beta _0+\beta _1\text {carat}_{n}+\beta _2\text {cut}_{n}+\dots +\beta _9\text {depth}_{n}+\varepsilon _n. \end{array}\right\} \end{aligned}$$

The *n* equations given in (1) could be expressed in form of matrices as

2 $$\begin{aligned} \left. \begin{pmatrix} \text {price}_1\\ \text {price}_2\\ \vdots \\ \text {price}_n \end{pmatrix}=\underbrace{\begin{pmatrix} 1&{}\text {carat}_{1}&{}\text {carat}_{2}&{}\dots &{}\text {carat}_{k}\\ 1&{}\text {cut}_{1}&{}\text {cut}_{2}&{}\dots &{}\text {cut}_{k}\\ \vdots &{}\vdots &{}\vdots &{}&{}\vdots \\ 1&{}\text {depth}_{1}&{}\text {depth}_{2}&{}\dots &{}\text {depth}_{k} \end{pmatrix}}_{\text {Design Matrix}}\begin{pmatrix} \beta _0\\ \beta _1\\ \vdots \\ \beta _k \end{pmatrix}+\begin{pmatrix} \varepsilon _1\\ \varepsilon _2\\ \vdots \\ \varepsilon _n \end{pmatrix}.\right\} \end{aligned}$$

Through algebraic operation, the *OLS* estimator of $\beta$ as described in (2) is given as:

$$\begin{aligned} \mathbf {\beta }=({\textbf{X}}^\prime {\textbf{X}})^{-1}{\textbf{X}}^\prime {\textbf{y}}. \end{aligned}$$

### Boosted classification and regression trees (BCARTs)

This is an ensemble learning method that combines multiple decision trees, but in a different way than RF. Instead of building multiple trees in parallel, it builds them sequentially, with each tree correcting the errors of the previous tree. It is often used for classification and regression tasks and has the ability to handle high-dimensional datasets thus candidate to this study. Tree boosting is a method of combining many weak learners (trees) into a strong classifier where: Each tree is created iteratively and the tree’s output *h*(*x*) is given a weight *w* relative to its accuracy. The ensemble output is the weighted sum:

$$\begin{aligned} {\hat{y}}(x)=\sum {_t}w_th_t(x). \end{aligned}$$

After each iteration each data sample is given a weight based on its misclassification i.e. the more often a data sample is misclassified, the more important it becomes. Here, the goal is to minimize an objective function:

$$\begin{aligned} O(x)=\sum {_i}l({\hat{y}}_i,y_i)+\sum {_t} \Omega (f_t). \end{aligned}$$

where:

- $l({\hat{y}}_i,y_i)$ is the loss function i.e. the distance between the truth and the prediction of the *ith* sample.
- $\Omega (f_t)$ is the regularization function i.e. it penalizes the complexity of the *tth* tree.

Further details on BCARTs could be obtained from^27^ and^28^.

### eXtreme gradient boosting (XGBoost)

XGBoost is a popular machine learning algorithm that uses decision trees to make accurate predictions. It works by combining many weak decision trees into a strong ensemble model that could handle complex relationships between input features and output labels. XGBoost has the ability to handle missing data and outliers, which are common in diamond datasets. The XGBoost algorithm tries to minimize the following objective function (loss function and regularization) *J* at step *t*:

$$\begin{aligned} J^{(t)}=\sum ^{n}_{i=1}L\left( y_i,{\hat{y}}_i^{t-1}+f_t(x_1)\right) +\sum ^{t}_{i=1}\Omega (f_i), \end{aligned}$$

where the first term contains the train loss function *L* (e.g. mean squared error) between real class *y* and output ${\hat{y}}$ for the *n* samples and the second term is the regularization term, which controls the the complexity of the model and helps to avoid overfitting^29^. It is observable that the XGBoost objective is a function of functions (i.e. *L* is a function of CART learners, a sum of the current and previous additive trees). To solve the above objective function, Taylor approximation is applied to transform the original objective function to a function in the Euclidean domain, in order to be able to use traditional optimization techniques. In XGBoost, the complexity is defined as:

$$\begin{aligned} \Omega (f)=\gamma T+\frac{1}{2}\lambda \sum ^{T}_{j=1}w^2_j, \end{aligned}$$

where *T* is the number of leaves, $\gamma$ is the pseudo-regularization hyperparameter, depending on each dataset and $\lambda$ is the *L*2 norm for leaf weights. Using gradients for second-order Taylor approximation of the loss function and finding the optimal weights *w*, the optimal value of objective function is:

$$\begin{aligned} J(t)=-\frac{1}{2} \sum ^{T}_{j=1} \frac{(\sum _{i\in I}g_i)^2}{\sum _{i\in I}h_i+\lambda }+\gamma T, \end{aligned}$$

where $g_i=\partial _{{\hat{y}}^{t-1}}L(y,{\hat{y}}^{t-1})$ and $h_i=\partial ^2_{{\hat{y}}^{t-1}}L(y,{\hat{y}}^{t-1})$ are the gradient statistics on the loss function, and *I* is the set of leaves. The XGBoost benefits from the shrinkage strategy in which newly added weights are scaled after every step of boosting (greedy algorithm) by a learning factor rate. This helps to diminish the effects of future new trees on every existing individual tree, thereby reducing the risk of over-fitting^30^.

### Support vector machine (SVM)

This is a supervised learning algorithm that finds the optimal hyperplane that separates the data into different classes. It is often used for classification tasks and is known for its ability to handle high-dimensional feature spaces and non-linear decision boundaries, a key characteristic with diamond dataset. SVM is a machine learning technique that works by identifying the optimal decision boundary that separates data points from different classes, and then predicts the class of new observations based on the said boundary. Kassambara^31^ observes that SVM could be used for two-class as well as multi-class classification problems. James et al.^16^ asserts that there is an extension of the SVM for regression (i.e. for a quantitative rather than a qualitative response), called *support vector regression*. Support vector regression seeks coefficients ($\beta _o,\beta _1,\dots ,\beta _p$) that minimize a different type of loss, where only residuals larger in absolute value than some positive constant contribute to the loss function.

Suppose that we have $n\times p$ matrix of data set, where samples belong to two linearly separable classes represented by $+1$ or $-1$, and suppose $g_i$ is the features vector. The, $(g_i,y_i) \in G \times Y, i=1,2,\dots ,n$ will be satisfied where $y_i \in ( +1,-1)$ is the target variable dichotomy in the p dimensional space. The aim is to classify the sample into one of the two classes and by extension find an SVM classifier that generalizes to a multi-class problem achieved by finding an optimal separating hyperplane^32^.

A separating hyperplane for the two classes is given as:

$$\begin{aligned} w\times g+b \ge 1, \text { when } y_i=+1, \text { and } w\times g+b \le -1, \text { when } y_i=-1, \end{aligned}$$

where *w* is the weight vector, *b* is the bias, and |*b*|/||*w*|| is the perpendicular distance to the hyperplane. The distance from the nearest point in each class to the hyperplane becomes 1/||*w*|| and 2/||*w*|| between the two classes after rescaling. The solution to the optimization problem is obtained by maximizing the margin:

$$\begin{aligned} \min _{w,b} ||w||^2, \text { subject to } y_i(w\times g+b)\ge 1,i=1,2,\dots ,n. \end{aligned}$$

In this study, we will employ one-vs-one multi-class classification in which the SVM classifier produces all possible pairs of binary classifications. Here, given that we have *k* classes where $k>2$, it follows that $\frac{k(k-1)}{2}$ binary classifiers are produced in the training step of the algorithm. Consequently, a sample in the test data-set is assigned the class label that is voted the most by the binary classifiers from the trained *one-vs-one* SVM.

### K-nearest neighbors (KNN)

K-nearest neighbors (kNN) is a non-parametric method used for classification and regression^33^. It makes predictions based on the *k* closest training examples in the feature space and is known for its simplicity and interpretability thus chosen for this study. Given a positive integer *K* and a test observation $x_0$, the KNN classifier first identifies the *K* points in the training data that are closest to $x_0$, represented by $\psi _0$. It then estimates the conditional probability for class *j* as the fraction of points in $\psi _0$ whose response values equal *j*:

$$\begin{aligned} P_r(Y=j|X=x_0)=\frac{1}{K}\sum _{i\in \psi _0}I(y_i=j). \end{aligned}$$

Lastly, KNN applies Bayes rule and classifies the test observation $x_0$ to the class with the largest probability^16^. The regression seeks to estimate $f(x_0)$ using the average of all the training responses in $\psi _0$, mathematically expressed as:

$$\begin{aligned} {\hat{f}}(x_0)=\frac{1}{K}\sum _{x_i\in \psi _0}y_i. \end{aligned}$$

### Random forests (RFs)

This is an ensemble learning method that builds multiple decision trees and combines their outputs to make a final prediction. It is often used for classification and regression tasks and is known for its high accuracy and ability to handle large datasets. Random Forest is an unpruned classification or regression tree ensemble produced by employing bootstrap samples of the training data and random feature selection in tree induction. The ensemble’s forecasts are summed (majority vote or averaging) to make a prediction. When creating these decision trees, a random sample of *m* predictors is picked as split candidates from the entire set of *p* predictors each time a split in the tree is evaluated. Only one of the m predictors could be used in the split. A fresh sample of *m* predictors is taken at each split, and typically we choose $m\approx \sqrt{p}$, i.e. the number of predictors considered at each split is approximately equal to the square root of the total number of predictors^16^. The random forest prediction is the most prevalent class among individual tree predictions in the *classification* setting. If there are *T* trees in the forest, the amount of votes a class *m* receives is:

$$\begin{aligned} v_m=\sum ^{T}_{t=1}I({\hat{y}}_t==m), \end{aligned}$$

where ${\hat{y}}_t$ is the prediction of the $t-th$ tree on a particular instance. The indicator function $I({\hat{y}}_t==m)$ takes on the value 1 if the condition is met, else it is 0. In a regression setting, the random forest’s forecast is the average of the individual trees’ predictions. If there are *T* trees in the forest, each making a prediction ${\hat{y}}_t$, the final prediction ${\hat{y}}_t$ is:

$$\begin{aligned} {\hat{y}}=\frac{1}{T}\sum ^{T}_{t=1}{\hat{y}}_t. \end{aligned}$$

### Multi-layer perceptron (MLP)

**Figure 2:**
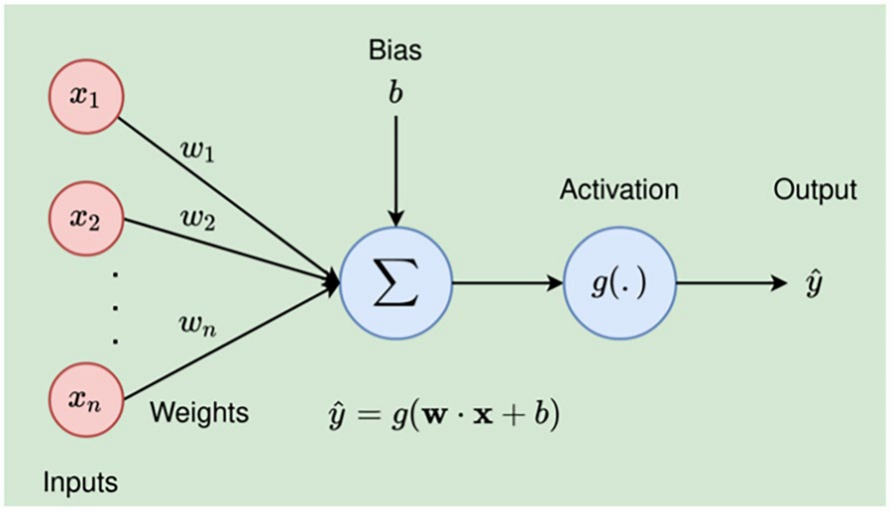
Neural network architecture.

This is a type of artificial neural network that consists of multiple layers of nodes, with each node connected to all nodes in the previous and next layers. It is often used for classification and regression tasks and is known for its ability to model complex relationships between inputs and outputs. MLP was chosen in this study for its ability to model non-linear relationships. It is inspired by the structure and function of the brain, which is usually called Artificial Neural Networks (ANN). To store information, the brain changes the connections between neurons. The neuron does not store information; instead, it enables signal transmission between neurons. The human brain is made up of a gigantic network of neurons. The neural network mimics the brain’s mechanism. While the human brain employs neuronal association, the neural network employs neuronal connection weights^34^. The information of the neural network is stored in the form of weights and biases as demonstrated in Fig. 2.

The input signals are multiplied by the weights before entering the node as shown in (3)

3 $$\begin{aligned} \text {price} = (w_1\times \text {carat})+(w_2\times \text {cut})+\ldots +(w_9\times \text {depth})+b ={\textbf {wx}}+b. \end{aligned}$$

The weighted sum could be expressed in matrix form:

$$\begin{aligned} {\textbf {price}}=\begin{bmatrix} w_1&w_2\dots w_9 \end{bmatrix}\begin{bmatrix} \text {carat}\\ \text {cut}\\ \vdots \\ \text {depth} \end{bmatrix}+\begin{bmatrix} b \end{bmatrix}. \end{aligned}$$

The output of the node (*y*) is processed using *activation function* (*g*) as shown in (4):

4 $$\begin{aligned} \hat{\text {price}}=\text {g} (v) =\text {g}({\textbf {w.x}}+b). \end{aligned}$$

**Figure 3:**
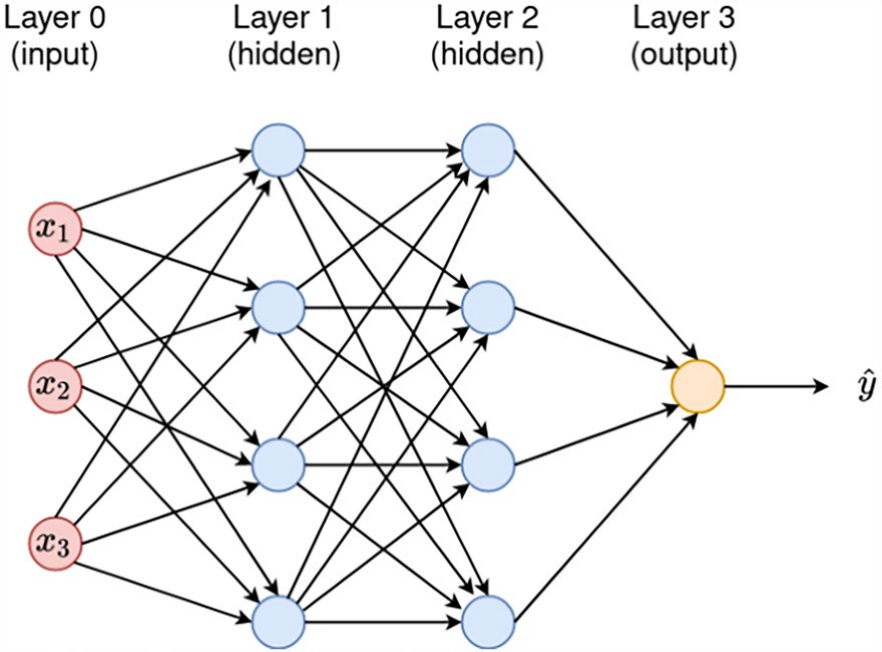
Multi-layer perceptron architecture.

It is important to note that MLP is defined by two or more hidden layers. According to^9^, the more hidden units there are in a network, the less likely it is to encounter a local minimum during training. Figure 3 shows a typical MLP network.

Input nodes merely relay the input signal; they do not compute the weighted sum or apply the activation function. Because they are not visible from outside the neural network, they are called hidden layers. In supervised learning, the learning rule trains the neural network to produce the proper output that has already been determined. The weights are initialized and the error is calculated accordingly. Then, the weights are adjusted to reduce the error. This procedure is repeated until the minimum error is attained. The systematic way of modifying the weights is known as the *Learning Rule*, as demonstrated by the *generalized delta rule* in (5).

5 $$\begin{aligned} \Delta w{ij} \leftarrow w_{ij}+\alpha \delta _i x_j, \end{aligned}$$

where

- $\delta _i=\phi ^\prime _i (v_i)e_i$, and $e_i$ is the error of node *i*, given by $e_i= d_i-y_i$. Here, $d_i$ describes the correct output while $y_i$ describes the observed output. The parameter $\phi ^\prime =\frac{d}{dx}$ is described as the activation function.
- $\Delta w_{ij}$ is the updated weight, $w_{ij}$ being the previous weight, $x_j$ is the output from node *j* ($j=1,2,3\dots$) while $\alpha$ is the learning rate ($0\le \alpha \le 1$).

The learning rate determines the extent to which weights are changed at every epoch. A high value of $\alpha$ indicates that the output gravitates around the expected solution while a low value shows that out fails to converge to the acceptable solution. Sigmoid, given in (6) will be used as the activation function.

6 $$\begin{aligned} \phi (X)=\frac{1}{1+e^{-X}}. \end{aligned}$$

Getting the first derivative of (6) expression (7) is obtained.

7 $$\begin{aligned} \phi ^\prime (x)=\phi (x)(1-\phi (x)). \end{aligned}$$

Multi-Layer Perceptron (MLP) has the following strengths:-

- The impediment of training multi-layers is solved by Back-propagation algorithm.
- Poor performance due to vanishing gradient is addressed by use of *Rectified Linear Unit* (ReLU), which is applied as the activation function.
- The vulnerability to over-fitting resulting from model complexity with additional hidden layers is solved by ‘Dropout’, i.e.training some of the randomly selected nodes rather than the entire network. Regularization is also used to prevent over-fitting by simplifying the architecture of MLP.
- The softmax activation function in the output layer helps to keep range between 0 and 1 which could be used as probabilities.

ReLU function gives us the maximum value between zero and a given input.

8 $$\begin{aligned} \phi (x)=\left\{ \begin{aligned}&x, ~~~~~x\ge 0\\ {}&0,~~~~~ x\le 0 \end{aligned}\right\} =\max (0,x). \end{aligned}$$

The derivative of ReLU function as described in (8) gives

$$\begin{aligned} \phi \prime (x)=\left\{ \begin{aligned}&1, ~~~~~x\ge 0\\ {}&0,~~~~~ x\le 0. \end{aligned}\right. \end{aligned}$$

The softmax activation function is given by

$$\begin{aligned} \phi ({{\textbf {z}}})_{i}=\frac{e^{z_{i}}}{\sum _{j=1}^{K}e^{z_{j}}}, \end{aligned}$$

where $\phi =\text { softmax }$, ${{\textbf {z}}}=$ input vector, $e^{z_{i}}=$ standard exponential function for input vector, $K =$ number of classes in the multi-class classifier, $e^{z_{j}}=$ standard exponential function for output vector.

The Adam optimization algorithm is a popular optimization method for training neural networks that builds upon two other optimization algorithms, stochastic gradient descent (SGD) and root mean squared propagation (RMSprop)^35^. The algorithm leverages exponential weighted moving averages, also known as leaky averaging, to estimate the momentum and second moment of the gradient. Specifically, Adam uses state variables that allow it to keep track of these estimates during the optimization process. The state variables are given as:

9 $$\begin{aligned} \begin{aligned} v_t&\leftarrow \beta _1 {\textbf{v}}_{t-1} + (1-\beta _1) {\textbf{g}}_t,\\ s_t&\leftarrow \beta _2 {\textbf{s}}_{t-1} + (1-\beta _2) {\textbf{g}}^2_t \end{aligned} \end{aligned}$$

$\beta _1$ and $\beta _2$ are two non-negative weighting parameters, typically chosen to be 0.9 and 0.999, respectively. It’s worth noting that the momentum term changes at a faster rate compared to the variance estimate. However, if we set the initial values ${\textbf{v}}_0={\textbf{s}}_0=0$, there will be a bias towards smaller values. To overcome this issue, we leverage on $\sum _{i=0}^{t-1} \beta ^i=\frac{1-\beta ^t}{1-\beta }$ to re-normalizing terms. The normalized state variables are expressed as follows:

10 $$\begin{aligned} \hat{{\textbf{v}}_t}=\frac{{\textbf{v}}_t}{1-\beta _1^t}\,\, \text {and}\,\, \hat{{\textbf{s}}_t}=\frac{{\textbf{s}}_t}{1-\beta _2^t}. \end{aligned}$$

With accurate estimations at our disposal, we proceed to formulate the update equations. Initially, we rescale the gradient similar to RMSProp to derive the following outcome:

11 $$\begin{aligned} {\textbf{g}}_t^{\prime }=\frac{\eta \hat{{\textbf{v}}_t}}{\sqrt{\hat{{\textbf{s}}_t}}+\epsilon }. \end{aligned}$$

Our update procedure differs from RMSProp in that it utilizes momentum $\hat{{\textbf{v}}_t}$ instead of the gradient itself. Additionally, there is a minor distinction in the rescaling method, which involves using $\frac{1}{\sqrt{\hat{{\textbf{s}}_t}}+\epsilon }$ instead of $\frac{1}{\sqrt{\hat{{\textbf{s}}_t}+\epsilon }}$. In practice, the former approach is generally more effective, which is why we deviate from the RMSProp method. In general, selecting the value of $\epsilon =10^{-6}$ strikes a suitable trade-off between numerical stability and accuracy. With all the necessary components in hand, we proceed to calculate the updates. The process is rather straightforward, and we arrive at a simple update equation given by:

12 $$\begin{aligned} {\textbf{X}}_t \leftarrow {\textbf{X}}_{t-1}-{\textbf{g}}_t^{\prime } \end{aligned}$$

The Adam algorithm combines the benefits of RMSprop, which adapts the learning rate ($\eta$) based on a moving average of the squared gradients, and SGD, which updates the weights in the direction of the negative gradient of the loss function. By maintaining estimates of both the first and second moments of the gradients, Adam is able to adapt the learning rate more dynamically and with less sensitivity to hyperparameter tuning than other optimization algorithms.

### Linear discriminant analysis (LDA)

This extends the LDA classifier to the case of multiple predictors. Here, the assumption is that ${\textbf{X}}=(\text {price}, \text {carat}, \ldots ,\text {depth})$ is drawn from a *multivariate normal* or *multivariate Gaussian* distribution $N(\mu k,\Sigma )$, with a class-specific multivariate mean vector and a common covariance matrix^16^. Chris^36^ postulates that LDA uses *Bayes Theorem* for classification which we could explain by noting that if we have *K* classes and we want to classify the qualitative response variable ${\textbf{Y}}=\text {cut}$ where there are $K=(Fair, Good, Very\,\,good, Premium, Ideal)$ possible distinct and ordered values derived as follows: Let $\pi _k$be the prior probability that a given randomly chosen observation comes from the $k^{\text {th}}$ class. Let $f_k(x)\equiv \,P_r(X=\text {price}|Y=Ideal)$ be the density function of ${\textbf{X}}$ for an observation from the $k^{\text {th}}$ class. $f_k(x)$ is relatively large if there is a high probability that an observation in the $k^{\text {th}}$ class has $X\approx \,x$ and $f_k(x)$ is relatively small if it is very unlikely that an observation in the $k^{\text {th}}$ class has $X\approx \,x$. Bayes Theorem states that:

13 $$\begin{aligned} P_r(Y=k|X=x)=\frac{\pi kf_k(x)}{\sum _{l=1}^K\pi lfl(x)}. \end{aligned}$$

Letting $p_k(x)=P_r(Y=k|X)$, we could simply plug in estimates of $\pi _k$ and $f_k(X)$ into the formula which could be generated with the software that then takes care of the rest. We refer to $p_k(x)$ as the *posterior* probability that an observation $X=x$ belongs to the $k^{\text {th}}$ class given the predictor value for that observation. Estimating $\pi _k$ is easy if we have a random sample of $Y's$ from the population but estimating $f_k(X)$ is more difficult. However, if we have an estimate for $f_k(x)$ then we could build a classifier that approximates the Bayes classifier.

By assuming that $X=(X_1,X_2\dots , X_p)$ is drawn from a multivariate Gaussian distribution, with a class specific mean vector and a common covariance matrix which we could write as $X\sim N(\mu ,\Sigma )$ to indicate that *p* has a multivariate Gaussian distribution. $E(X)=\mu \,$ is the mean of the *X* vector with *p* components and $\text {Cov}(X)=\Sigma$ is the $p\times p$ covariance matrix of ${\textbf{X}}$. Formally, the multivariate Gaussian density is given as

14 $$\begin{aligned} f(x)=\frac{1}{(2_\pi )^{\frac{p}{2}}|\Sigma |^{\frac{1}{2}}}\exp \left( -\frac{1}{2}(x-\mu )^T \Sigma ^{-1}(x-\mu )\right) . \end{aligned}$$

Plugging the density function for the $k^{\text {th}}$ class, $f_k(X=x)$ into equation (13), and applying some algebra we see that the Bayes classifier assigns $X=x$ to the class for which

$$\begin{aligned} \delta _k(x)=x^T\Sigma ^{-1}\mu _k-\frac{1}{2}\mu _k^T\Sigma ^{-1}\mu _k+\log \,\,\pi _k. \end{aligned}$$

is the largest. The Bayes decision boundaries represent the set of values *x* for which $\delta _k(x)=\delta _l(x)$. In other words for which $x^T\Sigma ^{-1}\mu _k-\frac{1}{2}\mu _k^T\Sigma ^{-1}\mu _k=x^T\Sigma ^{-1}\mu _l-\frac{1}{2}\mu _l^T\Sigma ^{-1}\mu _l$, for $k\ne l$ . The log $\pi _k$ term has disappeared because each of the three classes has the same number of training observations, thus $\pi _k$ is the same for each class. To estimate $\mu _1\dots ,\mu _k,\pi _1\dots ,\pi _k$ and $\Sigma$ we use similar conventions for the case where $p=1.$

**Figure 4:**
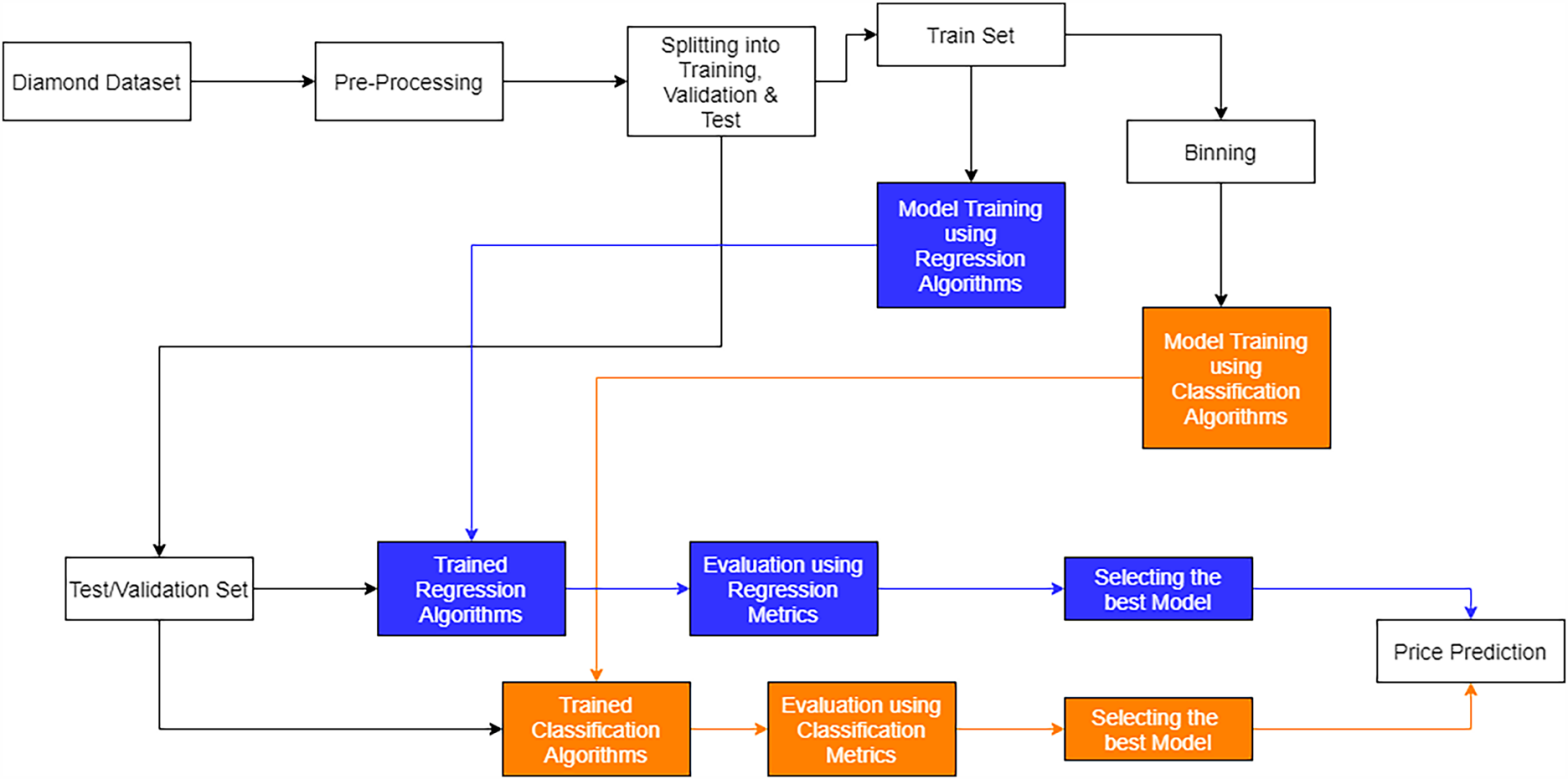
Overall modeling process.

The flowchart in Fig. 4 illustrates the overall modeling process.

Figure 4 depicts a flow chart showing the various options for achieving the final goal, which is diamond price forecast. While the blue coloring represents an existing technique to diamond pricing that uses machine learning, the orange shading represents the study’s alternative solution.

## Results

**Figure 5:**
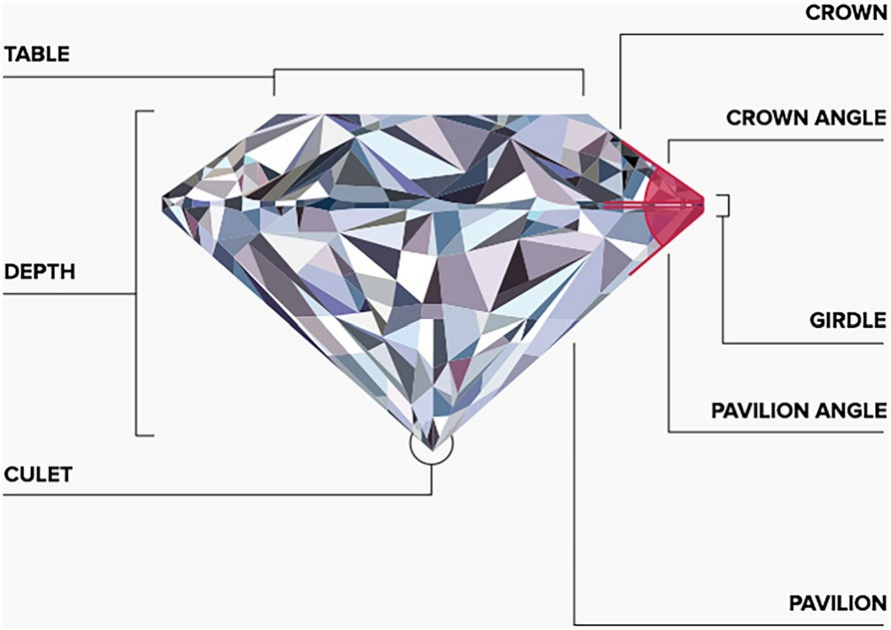
Diamond’s key features.

The various diamond features are defined based on Fig. 5 as follows:

- *Table* The top facet of a diamond is the table. If the table is out of proportion to the body of the jewel, it will negatively affect the brilliance and fire.
- *Depth* Depth of a diamond is measured by drawing an imaginary line between the center of the Top (table) and bottom (culet) facets.
- *Culet* The bottom point or facet of the diamond is the culet. A small, flat face at the culet is undesirable for a round cut diamond, but may provide positive characteristics for other shapes and styles.
- *Girdle* The girdle is typically a thin edge of the diamond where it could be held by the setting.
- *Pavilion Angle* Much like the crown angle, the pavilion affects sparkle and brilliance. When cut properly, the pavilion will emit the most sparkle through the top of the jewel. Cutting too shallow limits sparkle and makes the diamond seem glasslike. If the angle is too large the diamond will not emit optimal sparkle.

Table 1 describes the study variables.

**Table 1:** The study variables.

**Variable**	**Description**
Price	Price in US dollars (326–18,823)
Carat	Weight of the diamond (0.2–5.02)
Cut	Quality of the cut (Fair, Good, Very good, Premium, Ideal)
Color	Diamond color, from D (best) to J (worst)
Clarity	A measurement of how clear the diamond is (I1(worst), SI2, SI1, VS2, VS1, WS2, WS1, IF (best))
Table	Width of the top of diamond relative to widest point (43-95)
Depth	Total depth percent = z/mean( x,y) = 2*z/(x+y)(43-75)
x	Length in mm (0–10.74)
y	Width in mm (0–58.9)
z	Depth in mm (0–31.8)

**Figure 6:**
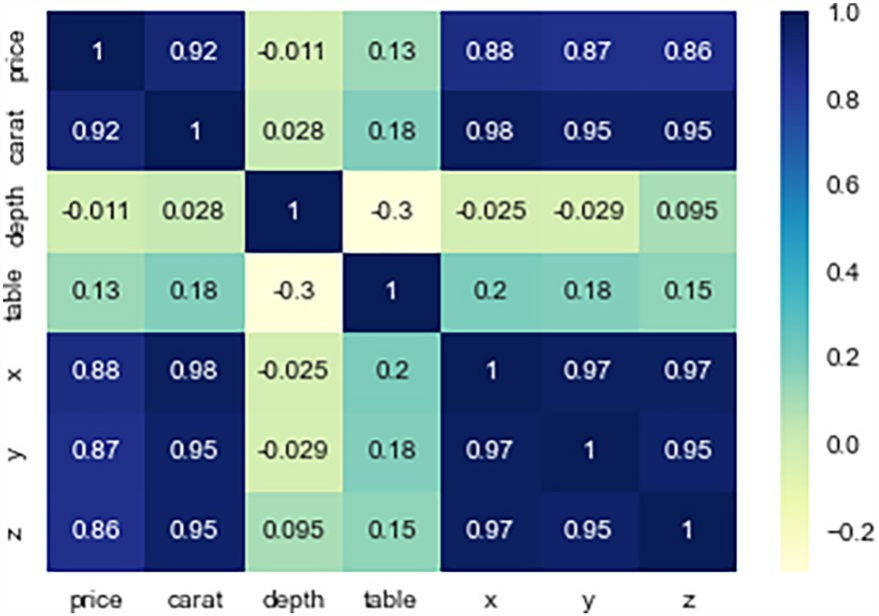
Correlation heatmap.

Figure 6 shows the correlation coefficients between the variables “price”, “carat”, “depth”, “table”, “x”, “y”, and “z”. The darker shades of blue indicate stronger positive correlation, while the darker shades of yellow and orange indicate negative correlation. From the heatmap, we could see that the carat weight has the strongest positive correlation with the price, followed by the dimensions x, y, and z. On the other hand, depth and table have weak to moderate negative correlation with price, which means that as these values increase, the price tends to decrease slightly. This is because these values could affect the visual appearance of the diamond, and buyers may be willing to pay less for diamonds with less desirable visual characteristics.

**Figure 7:**
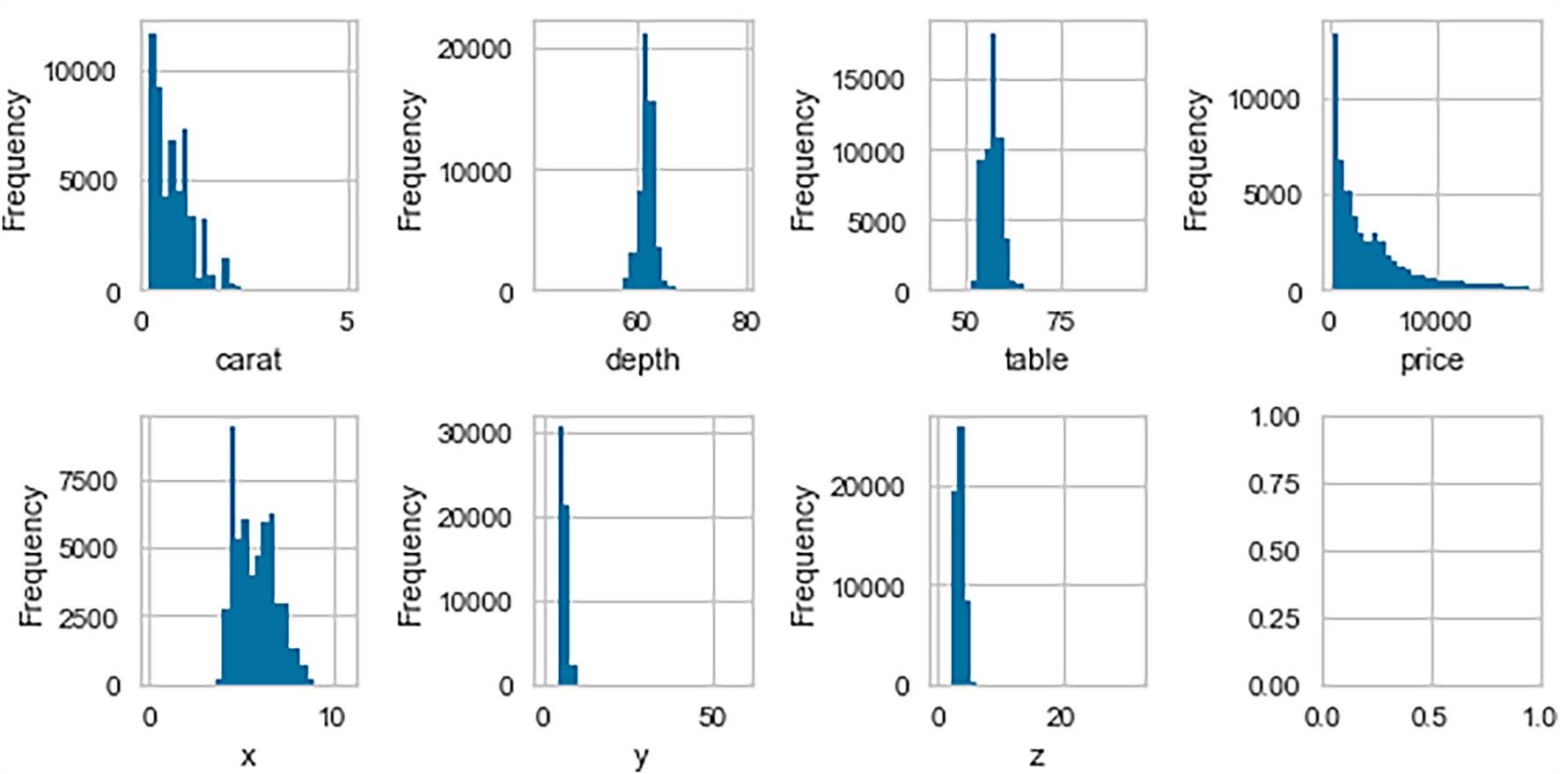
The histograms.

Figure 7 displays the distribution of the numerical variables in the “diamonds” dataset. The target variable, ‘price’, appears to be positively skewed, indicating that it deviates from normality. This deviation may impede the performance of algorithms that assume a normal distribution. Therefore, a transformation of the target variable is necessary to achieve normality and optimize the performance of the algorithms.

**Figure 8:**
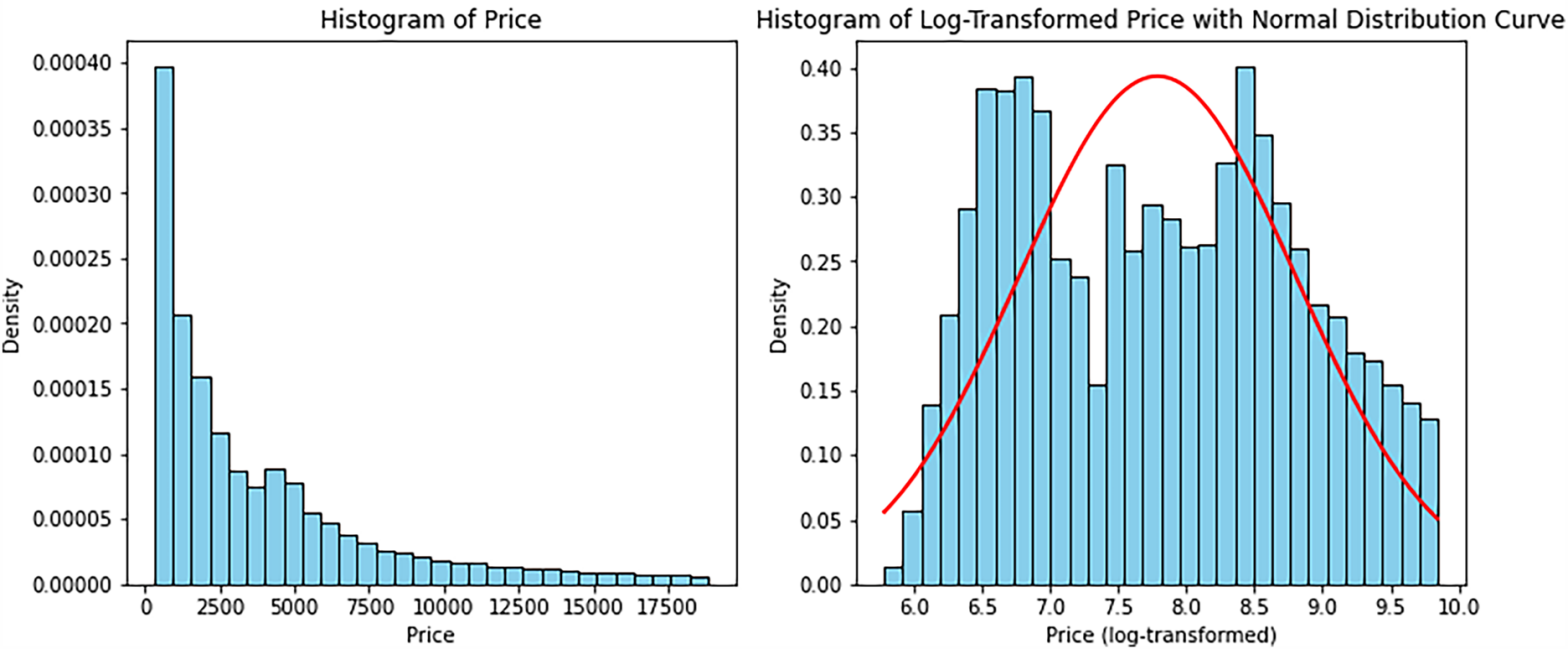
The Diamond’s logarithmic price transformation.

Figure 8 shows the distribution of the price variable before and after applying a logarithmic transformation. The left subplot shows the original distribution, which appears to be right-skewed. The right subplot shows the distribution after applying a logarithmic transformation, which results in a more symmetrical distribution. Applying a logarithmic transformation to the price variable proved to be useful in dealing with right-skewed price variable thus making the data more amenable to statistical analysis.

**Figure 9:**
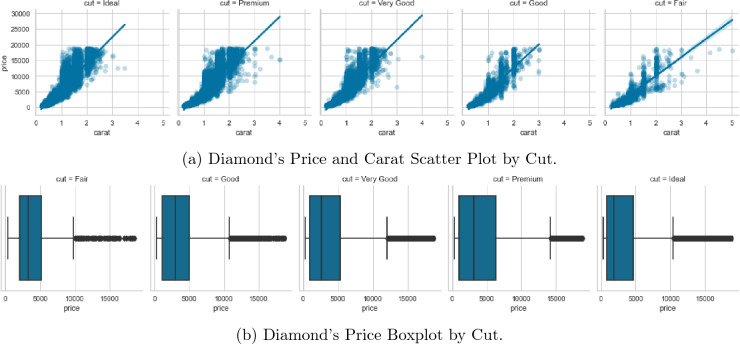
Diamond’s price, carat and cut relationships.

Figure 9a indicates that there is a positive linear relationship between carat weight and price. As the carat weight increases, the price of the diamond also tends to increase. The strength of the relationship between carat weight and price varies among different cuts of diamonds. The scatterplots for different cuts suggest that the positive linear association between carat weight and price is the strongest for the Fair and Ideal cuts, while it is weaker for the Good and Premium cuts. The spread of the data points is wider for higher carat weights, which suggests that there is more variability in the price of larger diamonds. There are some outliers in the data, particularly for the Fair cut, where some diamonds have a relatively high price for their carat weight compared to other diamonds in the same cut. The box plot in Fig. 9b shows the distribution of prices for each diamond cut category. From the plot, we could see that the median price of diamonds increases from Fair to Premium cut. The range of prices for each category varies, with Very Good and Premium cuts having the widest range and Fair cuts having the narrowest range. Additionally, there are outliers in each category, particularly in the Fair cut category. This suggests that the variability of diamond prices varies across different cut categories, with Premium cut diamonds having the most consistent prices.

## Discussion

In this study, we utilized both regression analysis and classification approaches to investigate the relationship between diamond prices and several variables, including categorical variables such as color, cut, and clarity. The primary aim was to develop an accurate predictive model for diamond prices. We used classification as an alternative model to the known regression way of diamond price prediction. Specifically, we binned the cut variable to predict mean prices for each cut category, aiming to identify patterns in the data that would enable accurate predictions of diamond prices based on the cut category. This was in contrast to the regression analysis, which considered the cut variable as a continuous variable. By employing the classification approach, we sought to improve the accuracy of our predictive model. To facilitate this analysis, the study used One Hot Encoding (OHE) to convert the categorical variables into a format compatible with machine and deep learning algorithms, a critical step in data preprocessing that improves model predictions and classification accuracy as demonstrated by^37^. Overall, the study aimed to develop a predictive model that accurately estimates diamond prices, utilizing both traditional regression analysis and a novel classification approach to improve accuracy.

The Random Forest (RF) model exhibited superior performance at default hyperparameters, with a Root Mean Squared Error (RMSE) of 1802.7425 for the train set and 108.8043 for the test set. The corresponding coefficient of determination ($R^2$) values were 100% and 81.1% for the train and test sets, respectively as shown in Fig. 11a. However, these results indicated a potential issue of overfitting in the model and a significant deviation of the line of best fit from the line of identity as depicted in Fig. 11a,b, respectively.

**Figure 10:**
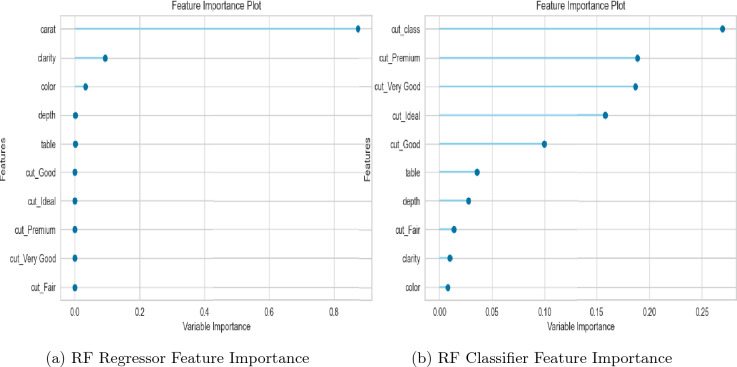
RF feature importance plots.

Initially, these findings were attributed to presence of outliers, missingness and multicollinearity, and lack of standardization in the data as revealed by Figs. 6, 7 and 9b. Outliers have a significant impact on the model’s performance, as they could skew the model’s predictions towards unrealistic values. Missingness, multicollinearity, and lack of standardization on the other hand are common issues that impact the accuracy and reliability of machine learning models. As part of the data preprocessing, the sudy addressed outliers, standardized the predictors, performed median imputation of missing values, and multicollinearity. The authors applied a correlation-based feature selection process in which a multicollinearity threshold of 0.9 was selected. This threshold was chosen to establish a stricter criterion for eliminating correlated variables and emphasize the precision of the model’s coefficients. As a result of this process, variables x, y, and z were identified as highly correlated with each other leading to their subsequent removal from the model as evidenced by their absence in Fig. 10a,b. IQR (Interquartile Range) method was applied to address outliers, identifying and eliminating values outside a certain range, typically 1.5 times the IQR. For normalizing the numerical features, standardization was employed, transforming them to have zero mean and unit variance, ensuring a consistent scale. To handle missing values in the numerical features, the authors adopted a robust strategy of imputing them with the respective feature’s median value, preserving the distribution and minimizing the influence of outliers.

**Figure 11:**
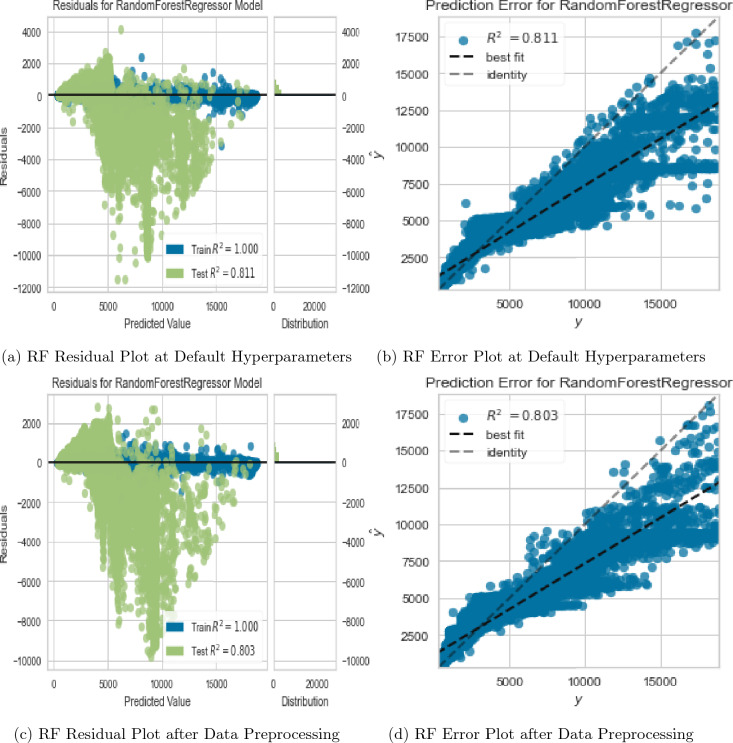
RF Regressor evaluation plots under different data & hyperparameter tweaks.

However, despite this progress, the model still exhibited signs of overfitting (Fig. 11c) and poor performance as evidenced by the significant deviation of the line of best fit from the line of identity in Fig. 11d. This indicated a need for a more robust model or further refinement of the RF regressor.

**Figure 12:**
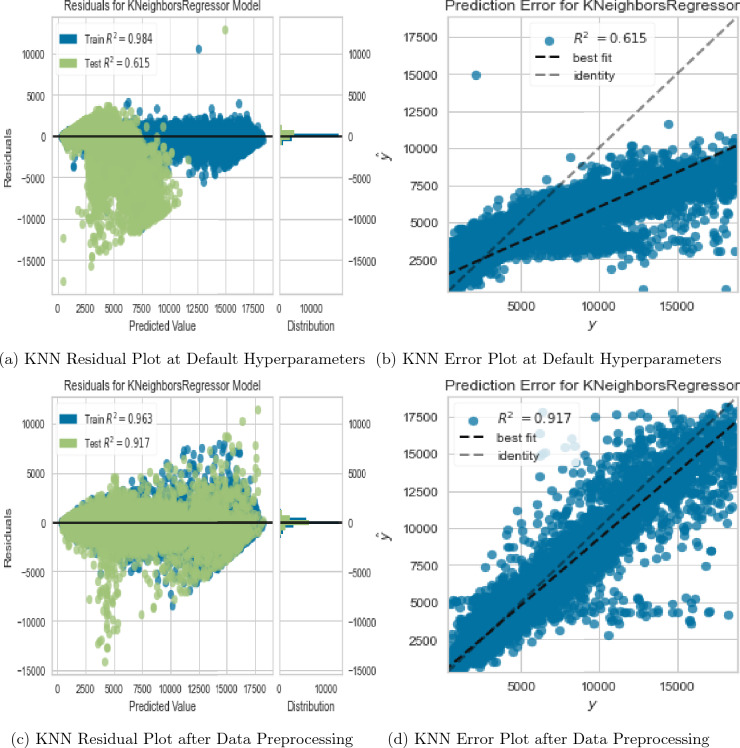
KNN regressor evaluation plots under different data & hyperparameter tweaks.

We evaluated the performance of the KNN regressor as an alternative to the RF regressor due to its outstanding performance after data preprocessing. Evidently from Fig. 12a,b, the performance of the KNN regressor with default hyperparameters was inferior in comparison to the RF regressor with default hyperparameters. To ensure a fair comparison, we applied the same data preprocessing steps to both models. We established that the KNN regressor outperformed the RF regressor on the preprocessed data as evidenced in Fig. 12c,d with train and test $R^2$ values of 96.3% and 91.7%, respectively. This improvement was attributable to two factors. First, addressing multicollinearity helped remove redundant features and allowed the KNN model to focus on the most important predictors, resulting in better performance due to its sensitivity to feature selection. Second, standardization helped ensure that features were on a similar scale, which was particularly important for the KNN model, as it relies on distance-based calculations.

Deviation of RF prediction errors from the identity line after data preprocessing (Fig. 11d) suggested that the RF model was overfitting the data, which meant it was capturing noise and random fluctuations in the data, rather than the underlying patterns. On the other hand, KNN model’s prediction errors after data preprocessing (Fig. 12d) were closer to the identity line, indicating that it was fitting the data better. To overcome this and improve the performance of both candidate models, we applied feature interaction and pseudo-labelling techniques to the preprocessed dataset.

Feature interaction involves creating new features by combining two or more existing features to capture any non-linear relationships between them. In their paper “Visualizing the Effects of Feature Interactions in Machine Learning Models”, Inglis et al.^38^ argue that feature interaction could help a machine learning model to capture complex interactions between features that may not be captured by individual features. Pseudo-labelling, on the other hand, involves using the trained machine learning model to generate labels for the unlabeled data and adding them to the training set^39^. Similarly, this could help increase the amount of labeled data and improve a model’s performance, according to^40^.

The diameter and volume of a diamond are important features in diamond price prediction because they are both strongly correlated with the carat weight of the diamond, which is a key factor that influences the diamond’s price. While carat weight is often considered one of the most important factors in determining the price of a diamond, the diameter and volume of the diamond could provide additional information about the diamond’s physical characteristics that could influence its price. Additionally, the diameter and volume of the diamond could provide information about the diamond’s cut quality. A well-cut diamond would reflect more light, which could increase its perceived value. The diameter and volume of the diamond could provide information about how effectively the diamond is cut, which could impact its price. Therefore, including the diameter and volume features in the diamond price prediction model would help to capture important information about the diamond’s physical characteristics and cut quality that would influence its price, in addition to the carat weight feature.

We calculated the ‘diameter’ of each diamond using the formula (carat $\times \,6.4)^{\frac{1}{3}}$. This formula is based on the assumption that the diameter of a diamond is proportional to its weight (measured in carats) raised to the power of $\frac{1}{3}$. The constant 6.4 is used to convert the weight to a diameter value that is in the same units as the other features. We derived the ‘volume’ of each diamond via feature interaction through multiplication of the diameter by depth and by table. This was based on the assumption that the volume of a diamond is proportional to its diameter, depth, and table size. The ‘depth’ feature represents the depth of the diamond in millimeters, while the ‘table’ feature represents the width of the top facet of the diamond as a percentage of its overall diameter, as shown in Fig. 5. Together, these two new features provided additional information about the size and shape of each diamond in the dataset, which proved to be useful for predicting its price.

**Figure 13:**
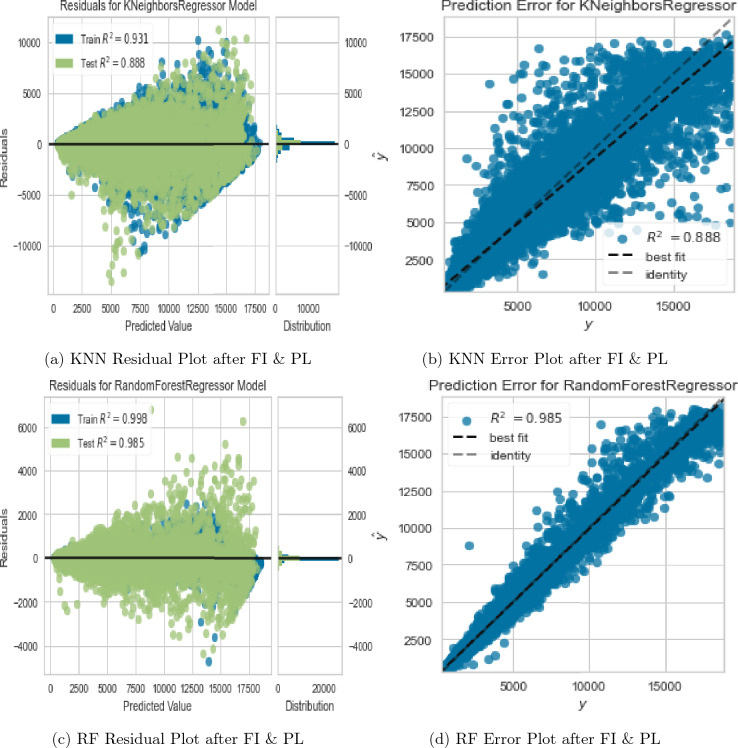
RF & KNN model performance evaluation plots after feature interaction (FI) and pseudo-labelling (PL).

After applying feature interaction and pseudo-labelling to the RF and KNN regressors, we observed a significant improvement and a slight drop in the RF and KNN model performances, respectively (see Fig. 13a). Specifically, the RF regressor’s RMSE decreased by 71.232%, and the R-squared value increased by 7.415% after feature interaction and pseudo-labelling. The KNN regressor’s RMSE increased by 16.547%, and its corresponding R-squared value decreased by 3.162%. The evaluation metrics for KNN regressor deteriorated, and its resultant prediction error plot depicted a significant deviation of the best fit line from the identity line as evident in Fig. 13b. This was an undesirable outcome, as it indicated that the KNN regressor inaccurately predicted the observed values, and there was systematic bias in it’s predictions after feature interaction and pseudo-labelling. On the other hand, the evaluation metrics for RF regressor improved, and its resultant prediction error plot showed an overlap of the best fit line and the identity line as evident in Fig. 13c,d. This implies that the RF regressor correctly captured the underlying relationship between the predictors and the target, and there was no significant evidence of any patterns or trends in the residuals that suggested the model was missing important information. This is an indication that the RF regressor was the best fit for the data.

**Figure 14:**
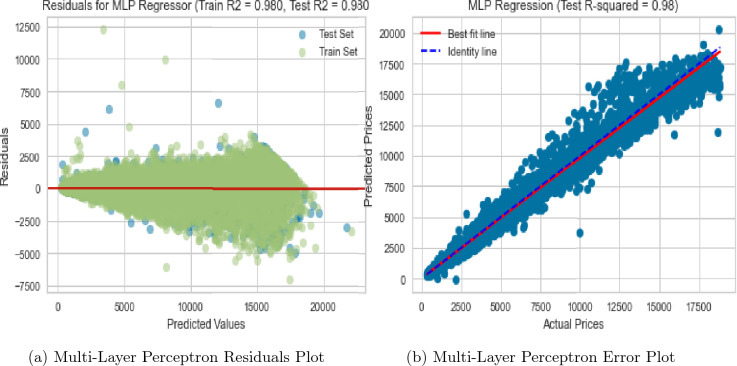
Multi-layer perceptron error and residuals plots.

In addition to the RF and KNN regressors, we also evaluated the performance of a Multi-layer perceptron (MLP) regressor on the dataset. The MLP regressor is a type of artificial neural network that could learn to model non-linear relationships between the predictors and the target variable. Our experiments showed that the MLP regressor achieved an RMSE of 563.742 and an R-squared value of 0.980 (Fig. 14a,b). These results suggest that the MLP regressor performed well on the dataset and was able to capture the complex non-linear relationships between the predictors and the target variable. Comparing the performance of the MLP regressor to that of the RF regressor, we could see that the RMSE of the MLP regressor is higher than that of the RF regressor, indicating a slightly worse performance in terms of the prediction error. The R-squared value of the RF regressor was higher than that of the MLP regressor, indicating a better fit to the data. Additionally, our experiments showed that the MLP regressor took 749 minutes, 59.9 seconds to train, while the RF regressor took only 5 seconds to train. This indicates that the RF regressor is not only a better fit for the dataset but also a more efficient algorithm in terms of computation time.

**Table 2:** Performance metrics of different models.

Model	RMSE	MSE	MAE	R2	Time (HH:MM:SS)
RF regressor	523.50	274052.77	253.80	0.985	00:00:05
MLP regressor	563.74	317802.79	286.31	0.980	12:29:59
XGBoost regressor	612.88	375621.89	323.08	0.972	00:00:09
Boosted dt regressor	711.31	505962.77	345.1120	0.968	00:00:04
Knn regressor	1346.65	1813463.53	728.61	0.887	00:00:03
Linear regression	1395.41	1947164.32	833.93	0.876	00:00:03
Support vector regression	3044.49	9268924.23	1632.91	0.421	00:00:50

Table 2 presents a comparison of the performance metrics among the selected models. The metrics include RMSE, MSE, MAE, $\text {R}^2$, and the time taken for model training. This table offers valuable insights into the relative performance of the models, allowing for an assessment of their effectiveness in predicting the target variable.

**Figure 15:**
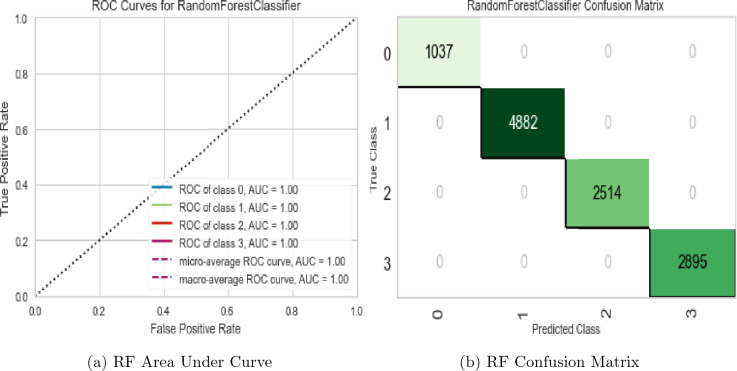
RF classifier evaluation plots.

In addition to our primary approach for diamond price prediction by regression, the study investigated a novel classification-based method utilizing a binned version of the ‘cut’ variable. The dataset was divided into five distinct classes based on the ‘cut’ values using the *‘pd.cut()’* function in Python and assigned a corresponding label to each bin. A classification model was then trained to predict the class of a diamond using its ‘cut’ value as the input feature. The price for diamonds in each class was approximated by utilizing the mean price of each respective class. To evaluate the performance of this classification-based approach, an ROC (Receiver Operating Characteristic) curve was constructed, which demonstrated that the RF classifier had a perfect discriminatory power with an area under the curve (AUC) of 1.00 (100%) as shown in Fig. 15a and supported by confusion matrix results as shown in Fig. 15b which confirms that there were no instances of misclassification.

The ROC curve is created by plotting the True Positive Rate (TPR) against the False Positive Rate (FPR) at different classification thresholds. TPR represents the proportion of true positive predictions (correctly classified positive instances) out of all actual positive instances, while FPR represents the proportion of false positive predictions (incorrectly classified negative instances) out of all actual negative instances. When the AUC is 1.00, it implies that the classifier achieves a TPR of 1 (perfectly identifies all positive instances) and an FPR of 0 (does not misclassify any negative instances). In other words, the classifier makes no false positive predictions and correctly identifies all positive instances, resulting in a straight line connecting the points (0,0) and (1,1) on the ROC curve. This indicates that the RF classifier was able to perfectly distinguish between positive and negative cases at all threshold settings and achieved highly accurate predictions of diamond prices based on the given features.

In summary, while the MLP regressor showed promising results and may be useful for predicting the price of diamonds in datasets with complex non-linear relationships, the RF regressor emerges as the optimal choice for diamond price prediction due to its superior performance in both regression and classification approaches. This conclusion is supported by several factors. Firstly, RF’s ability to handle non-linear relationships enables it to effectively capture the complex interactions and patterns within the dataset. Secondly, its robustness to outliers and noise ensures reliable predictions, enhancing its suitability for diamond price estimation. Additionally, RF’s efficient computation time allows for timely analysis of large datasets. Therefore, considering its exceptional performance in terms of R-squared, RMSE, AUC, and computational efficiency, the RF regressor stands out as the best fit for diamond price prediction, surpassing both the KNN and MLP regressors and classifiers. However, the MLP regressor still holds promise as an alternative approach for predicting diamond prices, particularly in datasets with complex non-linear relationships that are challenging to model using traditional machine learning algorithms like RF.

## Conclusion

Diamonds are precious stones that have been valued for centuries due to their unique properties, such as their hardness and brilliance. Recently, there has been an increasing interest in applying machine and deep learning algorithms to predict the price of diamonds based on their characteristics, such as carat weight, cut, color, and clarity. After evaluating the earlier mentioned machine and deep learning algorithms for predicting the price of diamonds based on their characteristics, including carat weight, cut, color, and clarity, we found that Random Forest (RF), Multi-layer perceptron (MLP) and K-Nearest Neighbors (KNN) emerged as the top-performing models.

The results of our experiments showed that feature interaction and pseudo-labelling had a positive effect on the performance of the RF regressor, as evidenced by a decrease in RMSE and an increase in R-squared value. Moreover, the resultant prediction error plot showed an overlap of the best fit line and the identity line, indicating that the RF regressor correctly captured the underlying relationship between the predictors and the target variable. However, the same techniques did not produce similar improvements in the performance of the KNN regressor. In fact, the RMSE increased, and the corresponding R-squared value decreased, indicating a systematic bias in the predictions of the KNN model. The resultant prediction error plot depicted a significant deviation of the best fit line from the identity line, which suggested that the KNN model inaccurately predicted the observed values.

In line with future research directions, it would be intriguing to assess the impact of different feature sets on model performance and explore techniques like regularization to enhance their effectiveness. Additionally, optimizing the binning process by investigating various bin sizes and boundaries, as well as considering other variables for binning, could be explored to further improve the accuracy of diamond price predictions. These avenues present exciting opportunities for future investigations in refining the model and expanding its capabilities.

On the account of our findings, it is evident that the classification-based approach utilizing a binned version of the ‘cut’ variable is a promising alternative to the primary regression-based approach for diamond price prediction. The RF classifier demonstrated a perfect discriminatory power with an AUC of 1.00, indicating highly accurate predictions. This study proposes the development and deployment of end-to-end GPU-accelerated data science workflows to increase processing power and handle complex algorithms such as MLP. Such workflows allow for rapid exploration, iteration, and deployment of work and could significantly reduce training time. For instance, our experiments have shown that the training of MLP took over 12 hours without these workflows, but with their implementation, the training time could significantly be reduced. The reduced training time could lead to more efficient data processing and analysis, making these workflows a valuable tool in machine learning. Further, the study recommends the creation and implementation of an interactive dashboard through which predictions generated by the RF model could be conveniently accessed, enabling instant decision-making for diamond industry players. This solution allows for the efficient consumption of RF model predictions, facilitating near real-time insights and prompt actions.

In conclusion, the findings of this study have significant implications for multiple stakeholders in the diamond industry, including manufacturers, retailers, and consumers. By adopting the Random Forest (RF) algorithm, manufacturers can enhance their diamond pricing models, leading to improved accuracy in price estimations. This, in turn, enables manufacturers to make informed decisions regarding production and inventory management. For retailers, leveraging RF’s predictive capabilities offers an opportunity to optimize pricing strategies, leading to competitive pricing, increased profitability, and enhanced responsiveness to market fluctuations. Furthermore, the integration of RF in diamond pricing models enhances transparency and trust in the industry, benefiting consumers by providing more accurate price estimations. With access to reliable information, consumers can make informed purchasing decisions and ensure fair value for their investments. The use of RF in the diamond industry therefore represents a valuable tool for all stakeholders, driving efficiency, profitability, and trust within the market.

## Data Availability

Supporting data and code are archived in Dryad 10.5061/dryad.wh70rxwrh. The supporting data and codes provided in electronic supplementary material is available online at https://datadryad.org/stash/share/C6GT0Srv2PTHitjNyS29EPgAk29hHu_s2RR6CyzVpeM.
